# Management and outcomes of pneumothorax in adult patients with Langerhans cell Histiocytosis

**DOI:** 10.1186/s13023-019-1203-5

**Published:** 2019-10-21

**Authors:** Pierre Le Guen, Sylvie Chevret, Emmanuelle Bugnet, Constance de Margerie-Mellon, Gwenaël Lorillon, Agathe Seguin-Givelet, Fanélie Jouenne, Dominique Gossot, Robert Vassallo, Abdellatif Tazi

**Affiliations:** 1 Assistance Publique-Hôpitaux de Paris, Hôpital Saint-Louis, Centre National de Référence des Histiocytoses, Service de Pneumologie, 1 Avenue Claude Vellefaux, 75475 Paris, Cedex 10 France; 2 Université de Paris, U1153 CRESS, Equipe de Recherche en Biostatistiques et Epidémiologie Clinique (ECSTRRA), Paris, France; 3 Assistance Publique-Hôpitaux de Paris, Hôpital Saint-Louis, Service de Biostatistique et Information Médicale, Paris, France; 4 Université de Paris, Assistance Publique-Hôpitaux de Paris, Hôpital Saint-Louis, Service de Radiologie, Paris, France; 5 Département Thoracique, Institut du Thorax Curie-Montsouris, Institut Mutualiste Montsouris, Paris, France; 6 Université de Paris, Assistance Publique-Hôpitaux de Paris, Hôpital Saint-Louis, Laboratoire de Pharmacologie Biologique, Paris, France; 7 Departments of Medicine, Physiology and Biomedical Engineering, Mayo Clinic, Rochester, MN USA

**Keywords:** Langerhans cell histiocytosis, Pneumothorax, Recurrence, Outcome, Management

## Abstract

**Background:**

Pneumothorax may recur during pulmonary Langerhans cell histiocytosis (PLCH) patients’ follow-up and its management is not standardised. The factors associated with pneumothorax recurrence are unknown.

**Methods:**

In this retrospective study, PLCH patients who experienced a pneumothorax and were followed for at least 6 months after the first episode were eligible. The objectives were to describe the treatment of the initial episode and pneumothorax recurrences during follow-up. We also searched for factors associated with pneumothorax recurrence and evaluated the effect on lung function outcome. Time to recurrence was estimated by the Kaplan Meier method and the cumulative hazard of recurrence handling all recurrent events was estimated. Univariate Cox models and Andersen-Gill counting process were used for statistical analyses.

**Results:**

Fourty-three patients (median age 26.5 years [interquartile range (IQR), 22.9–35.4]; 26 men, 39 current smokers) were included and followed for median time of 49 months. Chest tube drainage was the main management of the initial pneumothorax, which resolved in 70% of cases. Pneumothorax recurred in 23 (53%) patients, and overall 96 pneumothoraces were observed during the study period. In the subgroup of patients who experienced pneumothorax recurrence, the median number of episodes per patient was 3 [IQR, 2–4]. All but one recurrence occurred within 2 years after the first episode. Thoracic surgery neither delayed the time of occurrence of the first ipsilateral recurrence nor reduced the overall number of recurrences during the study period, although the rate of recurrence was lower after thoracotomy than following video-assisted thoracic surgery (*p* = 0.03). At the time of the first pneumothorax, the presence of air trapping on lung function testing was associated with increased risk of recurrence (hazard ratio = 5.08; 95% confidence interval [1.18, 21.8]; p = 0.03). Pneumothorax recurrence did not predict subsequent lung function decline (*p* = 0.058).

**Conclusions:**

Our results show that pneumothorax recurrences occur during an “active” phase of PLCH. In this observational study, the time of occurrence of the first ipsilateral recurrence and the overall number of pneumothorax recurrences were similar after conservative and thoracic surgical treatments. Further studies are needed to determine the best management to reduce the risk of pneumothorax recurrence in PLCH patients.

## Background

Pulmonary Langerhans cell histiocytosis (PLCH) is a rare diffuse cystic lung disorder that occurs mainly in young smokers of both genders [1]. In adults, it is frequently the only manifestation of the disease, but may also be a part of systemic disease [1].

Pneumothorax occurs in approximately 15–20% of PLCH patients [1]. It may be bilateral, recur during the course of the disease, and its management is not standardised [2]. The only one available series of 16 patients reported a high rate of pneumothorax recurrence (58%) after conservative treatment including chest tube drainage, as compared to no recurrence after thoracotomy (TCT) [3]. However, whether these results may be extrapolated to that of video-assisted thoracic surgery (VATS) - which is currently the main technical surgery performed for the surgical management of pneumothorax in general [4–6] , warrants further evaluation.

Although management of pneumothorax by thoracic surgical techniques is usually considered definitive, clinical experience suggests otherwise [7]. In addition, this reported lack of pneumothorax recurrence is inconsistent with observed pneumothorax relapse rates after thoracic surgery in other diffuse lung cystic disorders, i.e. lymphangioleiomyomatosis (LAM) and Birt-Hogg-Dubé (BHD) syndrome [8–10]_._

On the other hand, the factors that increase the risk of pneumothorax recurrence in PLCH patients remain poorly understood.

To address these issues, we analysed our cohort of PLCH patients to better characterize the patients who experienced pneumothorax, the treatment of the first episode and pneumothorax recurrences during follow-up. We also searched for factors associated with pneumothorax recurrence and evaluated the effect on lung function outcome.

## Methods

### Study design and subject selection

All patients 18 years of age or older with PLCH who were referred to the National Reference Centre for Histiocytoses between November 2003 and December 2015 were eligible for the study, provided they fulfilled the following inclusion criteria: 1) they experienced at least one pneumothorax; 2) information was available on pneumothorax management; 3) they were followed for at least 6 months after the first pneumothorax. Patients who experienced a pneumothorax long before diagnosis and unclearly related to PLCH have been excluded. The study period ended at 30th June 2016. Medical data of these patients had been prospectively registered and retrospectively analysed.

The diagnosis of LCH was either histologically confirmed by a biopsy of an involved site or was based on a typical lung high-resolution computed tomography (HRCT) pattern and the exclusion of alternative diagnoses [1].

The study was performed in accordance with the amended Helsinki Declaration and approved by the Institutional Review Board of the French Institute of Medical Research and Health (IRB number 17–395). All patients provided written informed consent for the use of their medical information for research.

### Data collection

Data on the patient demographics, smoking habits, cannabis consumption, clinical symptoms, clinical signs and LCH manifestations, systemic treatments received during the study, and lung function tests at the time of PLCH diagnosis and during follow-up were retrieved from the database. BRAF genotyping status was also recorded.

Stratification of LCH was performed according to the Histiocyte Society criteria and comprised either isolated lung involvement (single system disease, SS) or associated with other LCH manifestations (multisystem disease, MS) [11].

The type of procedures performed for the management of pneumothorax (including pleurodesis) was recorded for each episode. When several sequential procedures were performed to obtain pneumothorax resolution, the most invasive was designated as the main procedure for the pneumothorax episode.

For thoracic surgery, the type of intervention, i.e. VATS or TCT, was recorded. The resection or not of pulmonary cysts and/or bullae during the procedure was also noted. Finally, pleurodesis and its type (mechanical, chemical consisting in iodine or talc, or pleurectomy) were recorded.

HRCT scans performed at the time of the first pneumothorax were considered for the study. All these HRCT scans were analysed by a radiologist (C de M) and a chest physician (PLG), and were classified as previously described [12]. The presence of subpleural cysts, cysts > 1 cm or bullae was also recorded. Finally, the whole lung predominant HRCT cystic pattern (thick-, thin-, walled cysts and their size < 1 cm, > 1 cm) was also determined.

Lung volumes were evaluated by plethysmography and forced expiratory volume in one second (FEV_1_) and forced vital capacity (FVC) by the flow-volume curve. Diffusing capacity of carbon monoxide (D_LCO_) was measured using the single-breath method. The predictive values were determined as previously described [13]. Restriction was defined as a total lung capacity (TLC) < 80% of predicted values, air trapping as a residual volume (RV)/TLC ratio > 120% of predictive values and obstruction as a FEV_1_/FVC ratio < 70% [13]. Lung function outcome during follow-up was based on variations overtime (at least at 6 months interval) of FEV_1_ and/or FVC values of ≥15% as compared with baseline values. Thus, worsening of lung function was defined as a decrease of 15% or more in FEV_1_ and/or FVC [13].

BRAF genotyping was performed as previously described [14].

### Endpoints

The primary outcome was time to the first recurrence of pneumothorax. Secondary outcomes included: a) time to recurrence of all pneumothorax and restricted to the ipsilateral side; b) lung function worsening or development of new airflow obstruction during the study period.

### Statistical analysis

Summary statistics, i.e., median with interquartile range [IQR] or percentages were calculated. Time to recurrence from the date of resolution to the date of new pneumothorax, or last follow-up, was estimated by the Kaplan Meier method. We also estimated the cumulative hazard of recurrences that allowed the handling of all recurrent events.

Univariate Cox models were first used for the predictive analysis of time to the first recurrence after initial pneumothorax resolution. Then, we used the Andersen-Gill model that handles all recurrences (overall and restricting to the ipsilateral ones), taking into account the correlated but unspecified structure of the data [15]. All baseline predictors were considered as time fixed, except tobacco use that was introduced as a time-dependent covariate.

Comparison of pulmonary function tests across subsets used the nonparametric Wilcoxon test. Effect of recurrence of pneumothorax on the hazard of lung function worsening was analysed using Cox models, where it was included as a time-dependent covariate.

Statistical analyses were performed using SAS (SAS Inc., Cary, NC, USA) and R (https://www.R-project.org/) softwares. All tests were two-sided, with *p*-values < 0.05 denoting statistical significance.

## Results

### Study population

Among the 221 PLCH patients observed during the study period, 45 patients fulfilled inclusion criteria. Two patients were excluded because their pneumothorax occurred at 8.5 and 2 years, respectively, before PLCH diagnosis. The characteristics at diagnosis of the 43 patients retained in the study (median age 26.5 years [IQR, 22.9–35.4]; 26 men, 39 current smokers, among whom 14 also consumed cannabis) are outlined in Table 1.

**Table 1:** Characteristics of the patients at the time of diagnosis of PLCH

Characteristic	*N* = 43
Age, years, median, [IQR]	26.5 [22.9–35.4]
Male sex, n (%)	26 (60%)
Smoker, n (%)	39 (91%)
Ex-smoker	4 (9%)
Cannabis^a^, n (%)	14 (33%)
Asymptomatic	12 (28%)
Respiratory symptoms, n (%)	25 (58%)
Cough	17 (40%)
Dyspnoea	14 (33%)
NYHA II/III	13/1
Constitutional symptoms	11 (26%)
Histological diagnosis, n (%)	33 (77%)
Genotyping, n	22
*BRAF*^*V600E*^, n (%)	11 (50%)
Pneumothorax as initial manifestation	28 (65%)
Isolated PLCH, n (%)	30 (70%)
MS LCH, n (%)	13 (30%)
Non-pulmonary LCH localizations, n	
Bone	4
Pituitary stalk	9
Diabetes insipidus	9
Anterior hypophysis dysfunction	4
Skin	4
Peripheral lymph nodes	2
CNS	1
Liver	1
Lung function^b^, n	36
TLC, % of predicted, *n* = 33	94 [87–107]
VC, % of predicted, n = 35	80 [65–96]
RV/TLC, % of predicted, *n* = 29	141 [118–162]
FEV_1_, % of predicted, *n* = 36	75.5 [59–90.5]
FEV_1_/FVC %, *n* = 31	78 [70–84]
D_LCO_ % of predicted, *n* = 21	58 [53–71]
Restriction^c^, (%)	3 (9%)
Obstruction, (%)	7 (23%)
Air trapping, n (%)	20 (69%)

^a^all tobacco smokers

^b^evaluated before the first pneumothorax in 11 patients (median time interval of −7.0 months IQR [−21.8; − 2.8]) and after the pneumothorax within a median time of 4.5 months, IQR [2.0–10.5] in the 25 remaining patients

^c^restriction was defined as TLC < 80% of predicted; obstruction as FEV_1_/FVC < 70% and air trapping as RV/TLC > 120% of predicted

*Abbreviation definitions*: *PLCH* pulmonary Langerhans cell histiocytosis, *NYHA* New York heart association, *MS* multisystem, *CNS* central nervous system, *TLC* total lung capacity, *VC* vital capacity, *RV* residual volume, *FEV*_*1*_ forced expiratory volume in 1 s, *FVC* forced vital capacity, *D*_*LCO*_ diffusion of carbon monoxide

The diagnosis of LCH was histologically confirmed in 33 (77%) of the patients (lung biopsy *n* = 27; peripheral lymph node n = 2; skin *n* = 1; bone *n* = 1; oral mucosa *n* = 1; gut *n* = 1). LCH tissue genotyping was available for 22 patients: 11 (50%) harboured the *BRAF*^*V600E*^ mutation.

Thirty-six patients had a lung function measurement at a close time to PLCH diagnosis (median time 1.8 months [IQR, 0.4–5.9]). Lung HRCT was available at the time of the first pneumothorax episode for 28 patients (median 3.5 days [IQR 0.5–26.5]). Additional details on lung HRCT findings are provided in the Additional file 1.

### Characteristics and management of the first pneumothorax episode

A pneumothorax was the initial manifestation that led to PLCH diagnosis in 28 (65%) patients: in 19 (44%) patients it was the presenting manifestation of disease and in 9 (21%) patients, pneumothorax occurred within a median time of 2.1 months [IQR, − 2.7; − 1] before the diagnosis was established. Fifteen (35%) patients experienced their first pneumothorax after PLCH diagnosis, within a median time of 18 months [IQR, 7–57]. This first pneumothorax was right-sided in 21, left-sided in 17 and bilateral for 5 patients, accounting for a total of 48 hemithorax events.

Precise information on the treatment of the first pneumothorax was available for 42 patients. Because 5 of these patients had bilateral pneumothorax, a total of 47 procedures were performed (Table 2). Briefly, observation was the first treatment for 6 partial pneumothoraces (all small and well tolerated) and allowed the resolution of pneumothorax in 4 (67%) of these cases. Drainage was the initial treatment in 30 pneumothoraces and succeeded in 20 (67%) cases. Surgery was performed as the first procedure in 10 pneumothoraces and resulted in pneumothorax resolution in all cases, although one patient needed 2 successive VATS interventions (Table 2).

**Table 2:** Sequential procedures used for the management of the first episode of pneumothorax in PLCH patients^a^

Procedure	*N* = 47^b^
Observation^c^, n	6
• alone	4
• +drainage	1
• +drainage+ surgery + drainage +surgery	1
Aspiration + drainage, n	1
Drainage, n	30
• alone	20
• +surgery^d^	10
Surgery^e^, n	10

^a^information on the management of the first episode of pneumothorax was available for 42 patients

^b^because 5 patients had bilateral pneumothorax, a total of 47 hemithorax were treated

^c^well tolerated small pneumothorax in all cases

^d^two patients needed 2 successive VATS interventions

^e^one patient needed 2 successive VATS interventions

*Abbreviation definitions*: *PLCH* pulmonary Langerhans cell histiocytosis, *VATS* video-assisted thoracic surgery

Overall, considering each hemithorax separately and the fact that 4 patients needed 2 thoracic surgery interventions to obtain pneumothorax resolution, 25 surgical procedures were ultimately performed in 20/42 (48%) patients for the first episode of pneumothorax (Table 2). The 5 patients with bilateral pneumothorax required a surgical procedure (VATS on one side in 4 patients and a sternotomy with bilateral pleurodesis in one patient).

### Follow-up and pneumothorax recurrences

The median follow-up in the study was 49.1 months [IQR, 27.3–155]. At the time of the first pneumothorax, 37 patients were current-smokers and 6 ex-smokers; 22 patients were weaned for tobacco during the study period (7 patients were also weaned from cannabis).

**Fig. 1:**
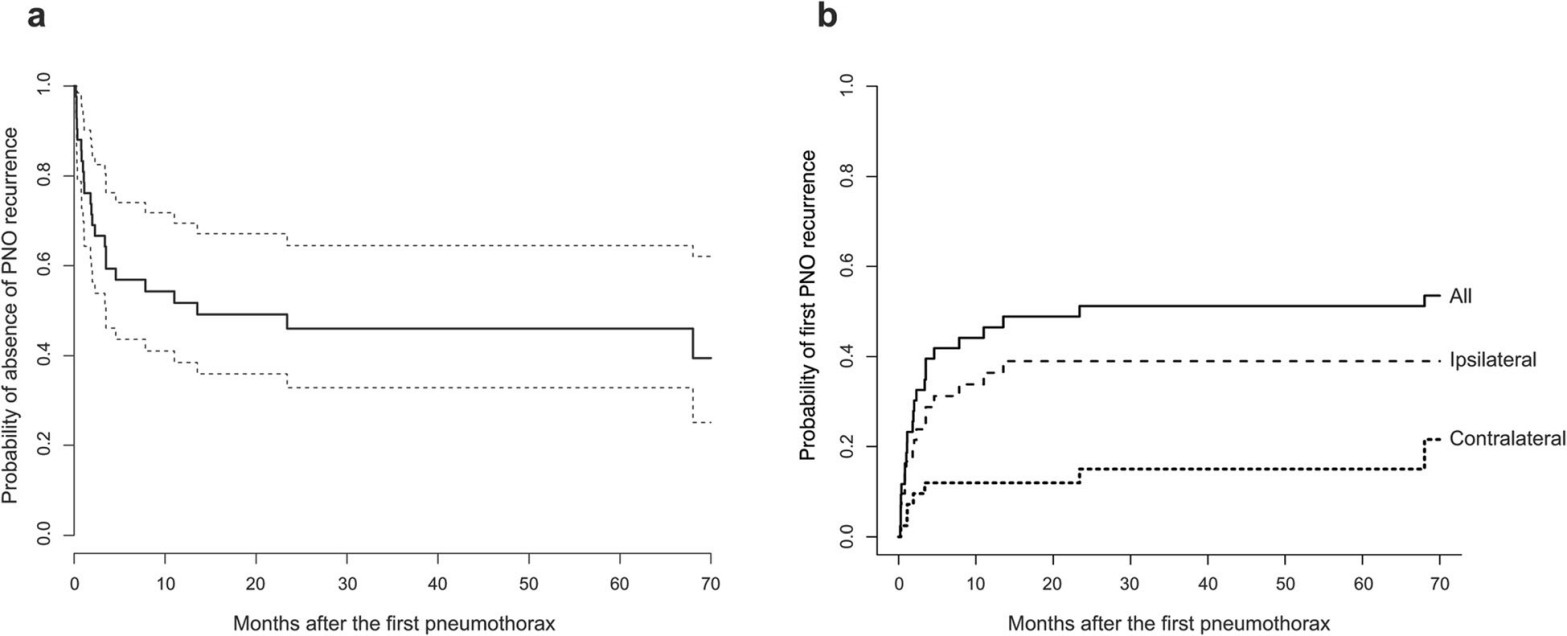
Distribution of time to PNO recurrence in the 43 PLCH patients. **a** Kaplan Meier estimate of the time to first recurrence, whatever the side of recurrence. Dashed lines indicate the limits of the 95% confidence interval. **b** Cumulative incidence of the first recurrence according to the side of pneumothorax recurrence. Note that all but one patient developed their first recurrence within 2 years after the first episode. The latter patient experienced a contralateral recurrence, 68 months after the first pneumothorax episode. *Abbreviation definitions:* PLCH, pulmonary Langerhans cell histiocytosis; PNO, pneumothorax

During the period of follow-up, 23/43 (53.5%) patients experienced at least one pneumothorax recurrence. The distribution of time up to the first recurrence is displayed in Fig. 1a.

The first pneumothorax recurrence occurred after a median time of 1.9 months [IQR, 0.8–4.0], and within 2 years in all but one patient; the latter experienced a contralateral recurrence, 68 months after the first pneumothorax (Fig. 1b). Of these 23 patients, 15 patients first experienced 16 ispsilateral recurrences (one presented recurrence of bilateral pneumothorax) and 8 patients had a recurrence involving the contralateral hemithorax. At 12 months, 52% (95% confidence interval (CI) 38.5–69.5%) of the patients were still free of any relapse.

During the study period, a total of 53 pneumothorax recurrences (41 ipsilateral) were observed, and were bilateral in 6 patients, accounting for 59 hemithorax events. Thus, with the 48 initial pneumothorax episodes, a total of 107 hemithorax events (96 pneumothoraces) were observed. The numbers of episodes per patient were as follows: 1 (*n* = 20), 2 (*n* = 9), 3 (*n* = 5), 4 (*n* = 4), 5 (n = 4) and 7 (*n* = 1). The median number of pneumothorax per patient was 2 [IQR, 1–3] in the whole study population, and 3 [IQR, 2–4] in the subgroup of 23 patients who experienced pneumothorax recurrence.

Information on the management of pneumothoraces was available for 106 hemithorax events: considering each hemithorax separately, 51 events were conservatively treated (observation *n* = 16; drainage *n* = 35), 2 were treated with medical pleurodesis (through the drainage chest tube), and thoracic surgery was performed for 53 episodes (VATS n = 35; thoracotomy *n* = 16; sternotomy *n* = 2), with 6 episodes needing 2 successive VATS for pneumothorax resolution (accounting for a total of 59 surgical procedures). Thus, excluding the patient who had a sternotomy for bilateral pneumothorax, 57 surgical procedures (41 VATS and 16 TCT) were performed during the study period. The type of TCT (available for 15/16 cases) consisted in limited axillary (*n* = 6), lateral (n = 5) and postero-lateral (*n* = 4) TCT. Pneumothorax resolution was obtained in 33/41 (81%) VATS and 15/16 (94%) TCT (*p* = 0.42) procedures. No statistical difference in baseline lung function parameters was observed comparing the patients whose pneumothorax was conservatively managed and those who needed thoracic surgery for obtaining pneumothorax resolution.

Table 3 details the different methods used for pleurodesis and their results for pneumothorax resolution. Mechanical abrasion associated with VATS resulted in the lowest rate of pneumothorax resolution, although it did not reach statistical significance.

**Table 3:** Results of pleurodesis performed in all PLCH patients treated surgically for their pneumothorax during the study^a^

Type of surgical procedure			
	VATS *N* = 40^b^	TCT *N* = 16	*P*-value (exact Fisher test)
Method of pleurodesis			
Mechanical alone	8	4	0.86^d^
Success, n (%)	5 (62.5%)	4 (100%)	
Chemical^c^	23	8	
Success, n (%)	19 (83%)	7 (87.5%)	
Apical pleurotomy	9	4	
Success, n (%)	8 (89%)	4 (100%)	
Overall success, n (%)	32 (80%)	15 (94%)	0.42

^a^each hemithorax is considered separately

^b^one patient had VATS without pleurodesis and was not included here

^c^chemical pleurodesis consisted in iodine (n = 9) or talc (*n* = 22) and could be associated to mechanical procedure

^d^comparing the 3 methods of pleurodesis

*Abbreviation definitions: PLCH* pulmonary Langerhans cell histiocytosis, *VATS* video-assisted thoracic surgery, *TCT* thoracotomy

### Ipsilateral recurrences

**Fig. 2:**
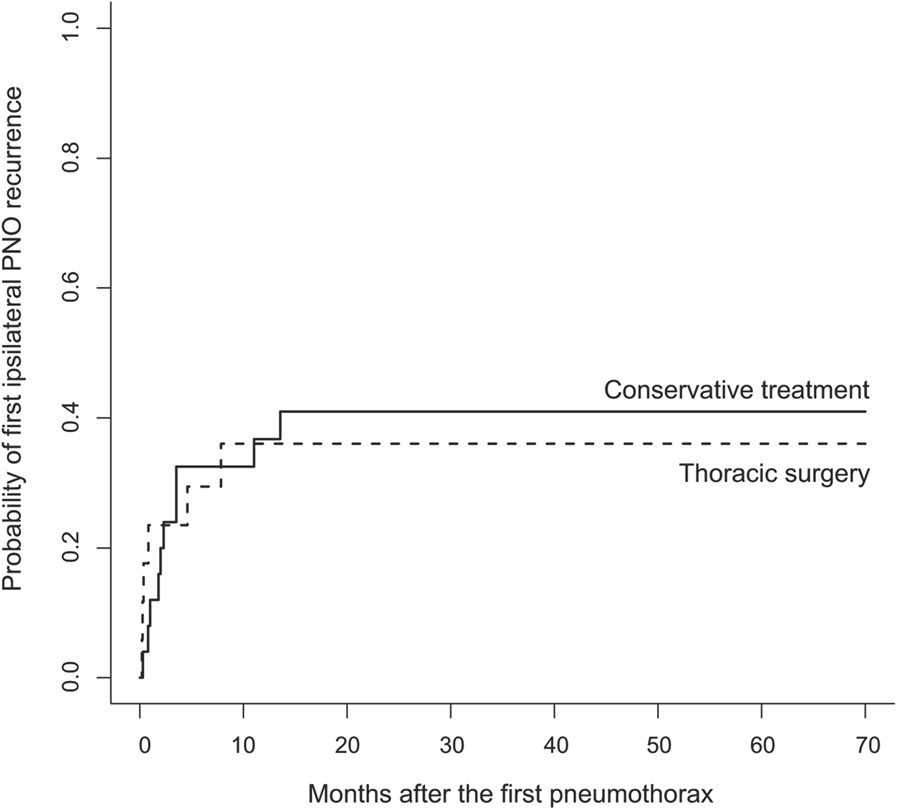
Probability of the first ipsilateral recurrence in the 43 PLCH patients, according to the treatment of the first pneumothorax episode. *Abbreviation definitions*: PLCH, pulmonary Langerhans cell histiocytosis; PNO, pneumothorax

Twenty patients experienced at least one ipsilateral recurrence. Thoracic surgery did not modify the cumulative incidence of the first ipsilateral pneumothorax recurrence that ocurred after a median time of 0.8 months [IQR, 0.4–4.6], compared to 2.1 months [IQR, 1.0–3.5] after drainage (*p* = 0.89, Fig. 2).

**Fig. 3:**
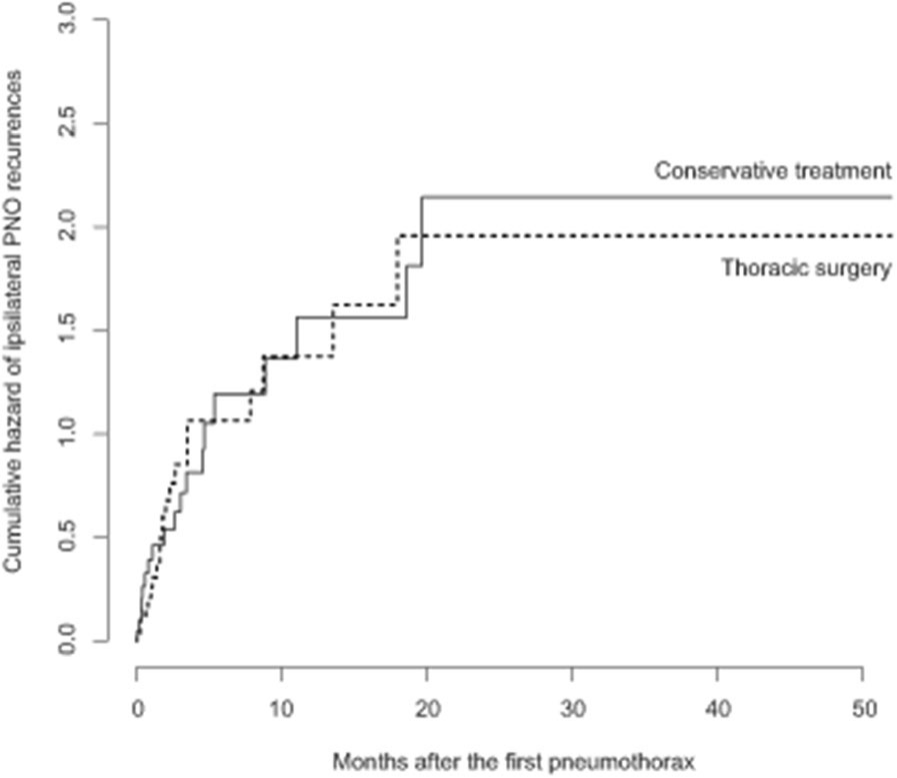
Cumulative hazard of pneumothorax ipsilateral recurrences in the 43 PLCH patients, according the treatment of the first episode. Note that all recurrences occurred within 2 years after the first pneumothorax episode. *Abbreviation definitions*: PLCH, pulmonary Langerhans cell histiocytosis; PNO, pneumothorax

These 20 patients experienced a total of 44 ipsilateral hemithorax recurrences (3 bilateral), within the 2 years after the first pneumothorax in all cases. Information on the management was available for 43 episodes: 21 (49%) recurrences occurred after conservative treatment (drainage *n* = 17, observation *n* = 4), and 22 (51%) after thoracic surgery (Additional file 1: Figure S1). The cumulative hazard all these events according to the treatment of the first episode is shown in Fig. 3.

When considering specifically the type of surgical procedure performed in the 43 patients of the study, 19 (54%) recurrences occurred after 35 VATS, whereas only 3 (19%) were observed after 16 TCT (*p* = 0.03).

The type of pleurodesis as well as the resection or not of cysts/bullae during surgical procedure did not modify the rate of ipsilateral recurrences. Additional details on results of surgical procedures performed are provided in the Additional file 1.

### Factors associated with pneumothorax outcomes

Table 4 shows the factors associated with pneumothorax ipsilateral recurrence, whatever the rank, based on Andersen-Gill univariable models. At the time of first PNO (that is, at inclusion in the study), air trapping was associated with increased hazard of pneumothorax recurrence (HR = 5.08, 95%CI [1.18; 21.8]; p = 0.03). Among the 22 patients with LCH tissue genotyping, the presence of the *BRAF*^*V600E*^ mutation was associated with a decreased hazard of pneumothorax recurrence (HR = 0.38, CI [0.17; 0.85]; *p* = 0.019). Smoking status over time did not influence the hazard of ipsilateral pneumothorax recurrences (HR = 0.73, 95%CI [0.38; 1.41]; *p* = 0.35). Similarly, cannabis consumption did not influence the risk of pneumothorax recurrence (Table 4).

**Table 4:** Univariate Andersen-Gill models: associated factors with any pneumothorax ipsilateral recurrences in PLCH patients during the study period

Characteristic	HR	CI 95%	*P* value
Time fixed: measured at baseline (first pneumothorax)			
Age	0.99	(0.96; 1.02)	0.65
Gender			
Female	1.00		
Male	1.21	(0.61; 2.42)	0.59
Tobacco smokers	1.00		
Ex-smokers	1.39	(0.65; 2.98)	0.35
Cannabis consumption, yes	1.71	(0.91; 3.2)	0.093
MS LCH	1.53	(0.8; 2.94)	0.20
TLC	0.98	(0.95; 1)	0.064
FVC	0.98	(0.96; 1)	0.020
FEV_1_	0.98	(0.96; 1)	0.041
FEV_1_/FVC	1.01	(0.97; 1.05)	0.67
Restriction	2.08	(0.84; 5.14)	0.11
Obstruction	0.38	(0.12; 1.28)	0.12
Air trapping	5.08	(1.18; 21.8)	0.029
HRCT nodular score	1.08	(0.98; 1.19)	0.11
HRCT cystic score	1.05	(0.93; 1.17)	0.43
Predominant:			
thick-walled cysts	1.76	(0.65; 4.77)	0.27
large cysts (> 1 cm)	0.20	(0.03; 1.43)	0.11
*BRAF* ^*V600E*^	0.38	(0.17; 0.85)	0.019
Time-dependent: measured during the sudy period			
Systemic treatment	0.68	(0.36; 1.28)	0.23
Smoking status^a^	0.73	(0.38; 1.41)	0.35
Surgery	1.00	(0.54; 1.87)	1.00
VATS	2.03	(1.00; 4.12)	0.050
Resection of cysts/bullae	1.58	(0.78; 3.2)	0.20
Chemical pleurodesis	1.48	(0.76; 2.88)	0.25
Mechanical abrasion	0.90	(0.48; 1.68)	0.75
Apical pleurectomy	1.22	(0.63; 2.36)	0.57

^a^persistent smoking vs. weaned from tobacco

*Abbreviation definitions: PLCH* pulmonary Langerhans cell histiocytosis, *MS* multisystem, *TLC* total lung capacity, *VC* vital capacity, *RV* residual volume, *FEV*_*1*_ forced expiratory volume in 1 s, *FVC* forced vital capacity, *D*_*LCO*_ diffusion of carbon monoxide, *HRCT* high resolution computed tomography, *VATS* video-assisted thoracoscopy

Nine (69%) of the 13 patients who had MS LCH at diagnosis experienced pneumothorax recurrences. The hazard of pneumothorax was not influenced by the MS nature of the disease (HR = 1.53, 95%CI (0.8; 2.94), *p* = 0.20) (Table 4).

Among surgically treated pneumothorax episodes, VATS was associated with increased hazard of subsequent recurrence (HR = 2.03, 95%CI [1.00; 4.12]; *p* = 0.050).

### Outcome of PLCH

Thirteen patients had received systemic treatments for their PLCH, which consisted in the following regimens: corticosteroids alone (*n* = 2); cladribine alone (*n* = 6); corticosteroids + vinblastine and with methotrexate (*n* = 1) and corticosteroids + vinblastine followed by cladribine (*n* = 4). Nine (69%) of these 13 patients experienced pneumothorax recurrence during their follow-up. Globally, all but two pneumothorax events occurred before the institution of systemic treatment. Only 2/9 patients experienced recurrent pneumothorax during or after systemic treatment.

Among the 30 untreated patients, 11 (37%) experienced pneumothorax recurrence during their follow-up. Considering systemic treatment as a time-dependent variable during the study period, the hazard of pneumothorax recurrences was decreased in patients who received systemic treatment (HR = 0.68, 95%CI, 0.36 to 1.28), although this decreased hazard was not statistically significant (*p* = 0.23) (Table 4).

**Fig. 4:**
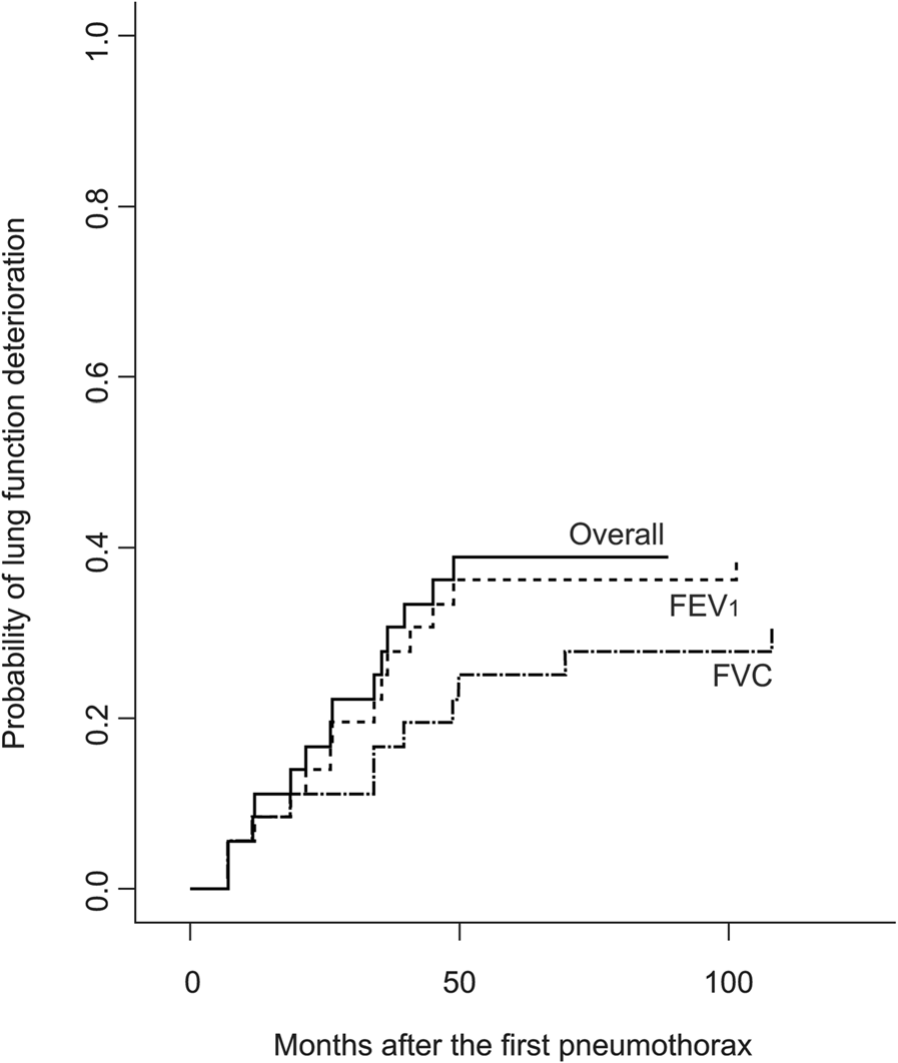
Probability of lung function deterioration in PLCH patients during the study period Over all means deterioration of either FEV_1_ or FVC or both. *Abbreviation definitions:* PLCH, pulmonary Langerhans cell histiocytosis; FEV_1_, forced expiratory volume in one second; FVC, forced vital capacity.

At the end of the study, 4 patients were under long term oxygen after a median time of 45.5 months following diagnosis. One patient had died following lung transplantation at 26 months after diagnosis. Thirty-six patients had at least one serial lung function measurement. As compared to baselines values, 14 (39%) patients deteriorated their FEV_1_ (*n* = 14; 39%) or FVC (*n* = 11; 31%) -including 11 who declined in both measures- during their follow-up (Fig. 4).

Seven patients developed a new-onset airflow obstruction, whereas it resolved in 3 of the 7 patients with baseline airflow limitation. At the end of the study, 11 (31%) patients had an airflow obstruction (median FEV_1_ 1600 ml [IQR, 1190–2420]; 44% of predicted [IQR, 33–55]). The hazard of lung function worsening after pneumothorax recurrence did not reach statistical significance (*p* = 0.058).

## Discussion

In this observational study of 43 PLCH patients experiencing a pneumothorax and followed for a median time of 49.1 months, we found the following salient results: 1) recurrent pneumothorax occurred in approximately half of the patients within 2 years following the initial pneumothorax, and were ipsilateral in three quarters of cases; 2) thoracic surgery did not modify the risk of pneumothorax recurrence; 3) in the univariate analyses, the presence of air trapping on lung function testing was associated with increased risk of pneumothorax recurrence.

The 50% rate of pneumothorax recurrence and the median number of 2 pneumothorax episodes in our study population were somewhat similar to those reported in a previous smaller series of 16 patients [3] and higher than that observed in a more recent larger study [2]. The rate of pneumothorax recurrences after PLCH is significantly lower than what was reported in LAM and BHD syndrome [8–10].

In this study, we also showed that the first pneumothorax recurrences occurred shortly, within a median time of 2 months, after the first episode. Furthermore, all but one recurrence occurred within the 2 years following the first pneumothorax, suggesting that these recurrences happened during an “active” phase of PLCH. The nodulo-cystic pattern observed in most patients for whom a lung HRCT was available at the time of the first pneumothorax episode is characteristic of recent onset PLCH [13].

The absence of reduction after thoracic surgery of the number of recurrences observed in our study appears different from what has been reported in other cystic lung diseases [8, 16], but these studies only dealt with the number of recurrences (assessed by a questionnaire), ignoring the time to recurrence in the analysis. Here, we used specific statistical methods that handle all times to recurrences (overall and in the same side that the previous episode) over the follow-up.

In this retrospective study, no recommendations were made for the management of pneumothorax, which was left to the discretion of the physicians in charge of the patients. As expected, the treatment of the first episode consisted in conservative (mainly drainage) treatment in the majority of patients and resulted in pneumothorax resolution in approximately 70% of cases [4]. Thoracic surgery was, however, eventually performed for the first episode in approximately half of the patients, including 30% of the patients who had conservative treatment initially. Because patients referred to the centre come from all over the country, these results roughly reflect the clinical practice in France.

The fact that thoracic surgery neither delayed the time of occurrence of the first ipsilateral recurrence nor reduced the overall number of recurrences during the study period, as compared with conservative treatment was unexpected. This result is apparently at odds with data of Mendez et al. who reported no relapse after thoracic surgery [3]. Actually, in that small study, all patients had been operated by TCT. In our study, the rate of ipsilateral recurrences was reduced after TCT as compared to VATS, suggesting that in case of pneumothorax ipsilateral recurrence, TCT might be considered. However, although, 2 meta-analyses also suggested a higher risk of recurrence after VATS as compared to TCT in patients with primary pneumothorax [17, 18], further studies are needed to confirm that it is also the case in PLCH patients. Our study covered a more recent period and thus depicts the current surgical practice favouring VATS [4–6]. In this regard, women with LAM and patients with BHD still experience 30–40% pneumothorax recurrence rate following surgical pleurodesis [8, 16]. The technique of pleurodesis used during surgical management of pneumothorax (including talc or pleurectomy) should be placed in perspective with possible complications during lung transplantation that may be a concern in a minority of these young patients in the long term [8, 19–21].

The presence of air trapping (i.e. increased RV/TLC ratio) at the time of inclusion in the study was associated with increased hazard of pneumothorax recurrence. Air trapping reflects the importance of bronchiolar impairment in PLCH which may be associated with distal airway pressure and increased risk of pneumothorax [1]. The reduced risk of pneumothorax recurrence among the patients whose PLCH lesions harboured the *BRAF*^*V600E*^ mutation should be taken with caution. In 2 previous studies, no association between BRAF status and PLCH outcome was identified [14, 22]. Since only half of the patients had BRAF genotyping, the finding of our study needs to be confirmed on a larger series.

Smoking status (including cannabis) over time did not reduce the risk of pneumothorax recurrence, although this might be different in a larger series of patients. It should be stressed, however, that smoking cessation was demonstrated to reduce the risk of subsequent lung function deterioration in PLCH patients in general [13].

This study has several limitations. Its retrospective design may have introduced a selection bias. The cohort studied is monocentric, although based on a national reference centre for this rare disease. Information on the size of chest tubes used and duration of pleural drainage was not available in most cases. Additionnally, this was an observational study, in which treatment decisions were left to the physician in charge of the patients, with potential confounding by indication bias, and thus the results regarding the effects of thoracic surgery should be taken with caution. The fact that several surgeons managed these patients with a surgical approach used not exactly the same between each surgeon may also have introduced another limitation, although increasing the external validity of the study. Finally, we did not perform multivariate analyses, because of the small number of events.

## Conclusions

Our results show that in PLCH patients, pneumothorax recurrences occur in approximately half of the patients within 2 years after the first episode, during an “active” phase of the disease. The presence of air trapping at the time of the first episode was associated with increased hazard of pneumothorax recurrence. Additional studies are needed to determine the best management to reduce the risk of pneumothorax recurrence in these patients.

## Supplementary information

**Additional file 1:** **Table S1.** Lung HRCT lesions and pattern at the first episode of pneumothorax in PLCH patients. **Table S2.** Ipsilateral pneumothorax recurrences after surgical procedures performed in PLCH patients during the study period. **Figure S1.** Sequential treatments of the first pneumothorax and the 44 ipsilateral recurrences observed in 20 PLCH patients who experienced ipsilateral recurrence.

## Data Availability

The data supporting the results reported in the current study are available from the corresponding author upon request.

## Abbreviations

BHD: Birt-Hogg-Dubé syndrome
CI: Confidence interval
FEV_1_: Forced expiratory volume in 1 s
FVC: Forced vital capacity
HRCT: High resolution computed tomography
IQR: Interquartile range
LAM: Lymphangioleiomyomatosis
LCH: Langerhans cell histiocytosis
PLCH: Pulmonary Langerhans cell histiocytosis
TCT: Thoracotomy
TLC: Total lung capacity
VATS: Video-assisted thoracic surgery
