# Supplementary material for: Management and outcomes of pneumothorax in adult patients with Langerhans cell Histiocytosis
Source: Orphanet J Rare Dis. 2019 Oct 21;14:229. doi: 10.1186/s13023-019-1203-5 (PMC6805357; doi:10.1186/s13023-019-1203-5)
Supplement: Supplementary file 1 — Additional file 1: Table S1. Lung HRCT lesions and pattern at the first episode of pneumothorax in PLCH patients. Table S2. Ipsilateral pneumothorax recurrences after surgical procedures performed in PLCH patients during the study period. Figure S1. Sequential treatments of the first pneumothorax and the 44 ipsilateral recurrences observed in 20 PLCH patients who experienced ipsilateral recurrence. [file 13023_2019_1203_MOESM1_ESM.pdf]

Additional file 1: Supplementary material

Management and Outcomes of Pneumothorax in Adult Patients with Langerhans Cell Histiocytosis.

Pierre Le Guen, Sylvie Chevret, Emmanuelle Bugnet, Constance de Margerie-Mellon, Gwenaël Lorillon, Agathe Seguin-Givelet, Fanélie Jouenne, Dominique Gossot, Robert Vassallo, Abdellatif Tazi

Table S1. Lung HRCT lesions and pattern at the first episode of pneumothorax in PLCH patients

	Nodulo-cystic pattern	Cystic pattern	Cystic score Median, IQR	Predominant thick-walled cysts	Predominant thin-walled cysts	Predominant cysts >1cm	Presence of subpleural cysts
N=28	22	6	8.0 [IQR, 6-12]	14	14	6	27
Without recurrence N=16	12	4	8.5 [IQR, 6-12]	8	8	5	15
With recurrence N=12	10	2	7.5 [IQR, 7-10]	6	6	1	12

Abbreviation definitions: PLCH, pulmonary Langerhans cell histiocytosis; HRCT, high resolution computed tomography; IQR, interquartile range.

Table S2. Ipsilateral pneumothorax recurrences after surgical procedures performed in PLCH patients during the study period

		Pleurodesis									
		Mechanical		Chemical*		Both		Pleurectomy**		Total	
		n=9		n=23		n=6		n=12		n=50†	
All procedures	Resection[§] yes/no	7 (78%)	2 (22%)	9 (39%)	14 (61%)	3 (50%)	3 (50%)	5 (42%)	7 (58%)	24 (48%)	26 (52%)
	Recurrence, n (%)	2 (29%)	1 (50%)	4 (44%)	9 (64%)	0	0	1 (20%)	4 (57%)	7 (29%)	14 (54%)
	Total recurrences	3 (33%)		13 (56%)		0		5 (42%)		21 (42%)	
		n=5		n=21		n=0		n=8		n=34†	
VATS	Resection (yes/no)	4 (80%)	1 (20%)	8 (38%)	13 (62%)	NA	NA	3 (38%)	5 (62%)	15 (44%)	19 (56%)
	Recurrence, n (%)	2 (50%)	1 (100%)	3 (38%)	8 (62%)	NA	NA	1 (33%)	3 (60%)	6 (40%)	12 (63%)
	Total recurrences	3 (60%)		11 (52%)		NA		4 (50%)		18 (53%)	
		n=4		n=2		n=6		n=4		n=16	
TCT	Resection yes/no	3 (75%)	1 (25%)	1 (50%)	1 (50%)	3 (50%)	3 (50%)	2 (50%)	2 (50%)	9 (56%)	7 (44%)
	Recurrence, n (%)	0	0	1 (100%)	1 (100%)	0	0	0	1 (50%)	1 (11%)	2 (29%)
	Total recurrences	0		2 (100%)		0		1 (25%)		3 (19%)[¶]	

*chemical pleurodesis consisted of iodine or talc.
**apical pleurectomy whatever other associated procedure.
§resection of lung cysts/bullae.

†one patient did not have pleurodesis.
¶p=0.03 compared to VATS.

Abbreviation definitions: PLCH, pulmonary Langerhans cell histiocytosis; VATS, video-assisted thoracoscopy; TCT, thoracotomy.

Figure S1. Sequential treatments of the first pneumothorax and the 44 ipsilateral recurrences observed in 20 PLCH patients who experienced ipsilateral recurrence.

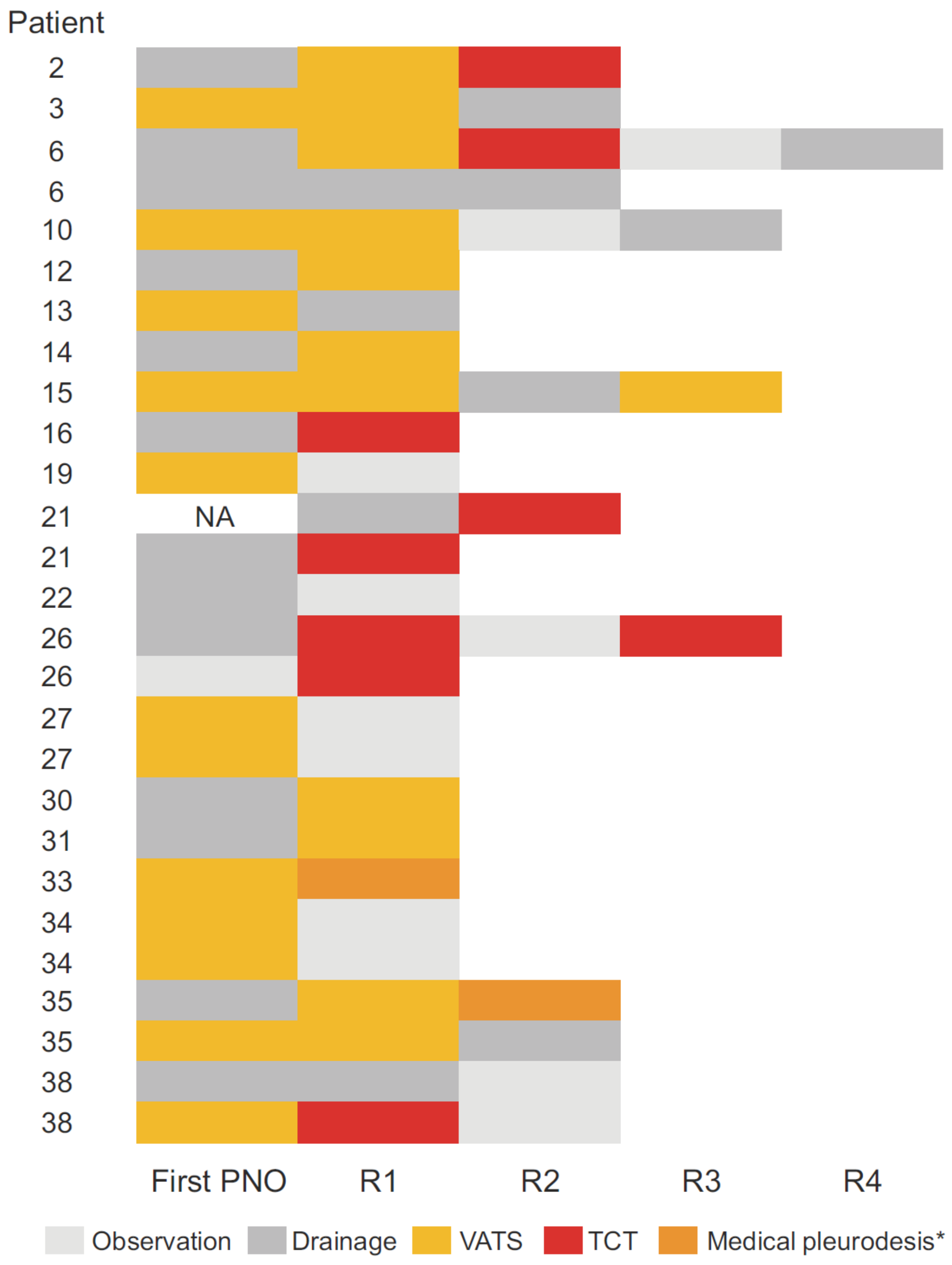

* Medical pleurodesis was performed through the drainage chest tube.

Abbreviations: PLCH, pulmonary Langerhans cell histiocytosis; NA, not available; PNO, pneumothorax; R, recurrence; VATS, video-assisted thoracoscopy; TCT, thoracotomy.
